# How big is Big Data? A comprehensive survey of data production, storage, and streaming in science and industry

**DOI:** 10.3389/fdata.2023.1271639

**Published:** 2023-10-19

**Authors:** Luca Clissa, Mario Lassnig, Lorenzo Rinaldi

**Affiliations:** ^1^Department of Physics and Astronomy, University of Bologna, Bologna, Italy; ^2^National Institute for Nuclear Physics, Bologna, Italy; ^3^CERN, Genève, Switzerland

**Keywords:** big data, data production, data volumes, data storage, streaming data

## Abstract

The contemporary surge in data production is fueled by diverse factors, with contributions from numerous stakeholders across various sectors. Comparing the volumes at play among different big data entities is challenging due to the scarcity of publicly available data. This survey aims to offer a comprehensive perspective on the orders of magnitude involved in yearly data generation by some public and private leading organizations, using an array of online sources for estimation. These estimates are based on meaningful, individual data production metrics and plausible per-unit sizes. The primary objective is to offer insights into the comparative scales of major big data players, their sources, and data production flows, rather than striving for precise measurements or incorporating the latest updates. The results are succinctly conveyed through a visual representation of the relative data generation volumes across these entities.

## 1. Introduction

In the last twenty years, we have witnessed an unprecedented and ever-increasing trend in data production. Hilbert and López (2011) date the rise of this phenomenon back to 2002, marking the onset of the digital age. Indeed, the transition from analog to digital storage devices dramatically augmented the capacity for data accumulation, thereby ushering in the *Big Data* era.

The term “big data” was first coined in 1990s (Mashey, 1998; Lohr, 2013) and it is typically used to denote datasets whose size exceeds the potential to manipulate and analyze them within reasonable time limits (Snijders et al., 2012). However, the expression does not refer to any specific storage size but assumes a more profound meaning that extends far beyond the sheer volume of data points. In fact, big data embrace a broad spectrum of data sources including structured, semi-structured and, predominantly, unstructured data (Dedić and Stanier, 2016). Although multiple connotations have been attributed to the concept of big data over the years, a commonly shared definition revolves around the so-called *5 Vs* (Jain, 2016):

- Volume: the actual quantity of generated data is large, in the order of magnitude of terabytes and petabytes (Sagiroglu and Sinanc, 2013). More generally, it indicates volumes that are too large and complex to be handled with conventional data storage and processing technologies;
- Variety: the data can originate from a multitude of sources and types, including sensors, social media, log files and more, and it covers a diverse range of formats like text, images, audio or video;
- Velocity: the data are generated and/or processed at high rates (Kitchin and McArdle, 2016), typically nearly real-time;
- Value: the data must carry valuable information that provides business value and profitable insights (Uddin et al., 2014). In a scientific context, this translates to information that contributes to the advancement of human knowledge;
- Veracity: the data sources must be reliable and generate high-quality data that can yield value (Schroeck et al., 2012; Onay and Öztürk, 2018).

However, the community has yet to reach a full consensus on the definition of big data (Grimes, 2013; Kitchin and McArdle, 2016), with some authors advocating for a shift in characterization from the intrinsic data properties to the techniques employed for acquisition, storage, circulation and analysis (Balazka and Rodighiero, 2020).

### 1.1. Big data origins and trends

The rise of the big data era is not solely due to advancements in storage capabilities. In fact, numerous other factors have significantly amplified data generation. The widespread adoption of the internet and the evolution of computer technologies have expanded processing capabilities and simplified data access, catalyzing further data generation. Consequently, there has been an increased contribution from various stakeholders, including tech giants, traditional industries, governments, healthcare institutions, scientific collaborations, and others. Moreover, the emergence of *smart* everyday objects designed for both receiving and producing data exponentially increased individual contributions to the overall data produced. Modern objects are often equipped with technologies that enable data collection and sharing via a network, commonly referred to as Internet of Things (Ashton et al., 2009). This phenomenon has further fueled the data production rate. For example, sensors measuring status and operation are now commonly used in industrial machinery and household appliances, simplifying their control and enabling automated maintenance. This trend has also extended to the personal items market, with tech companies increasingly investing in wearable devices such as watches and glasses. These objects allow users to stay connected to a rapidly evolving environment, track personal progress, and explore the world through virtual reality in unprecedented ways. Furthermore, digitization solutions are being explored to address the emerging challenges of our times. For instance, consider the urgent need for modernization of institutional processes posed by the pandemic. The massive spread of the infections has required unprecedented access to health assistance. However, the inability to scale up services and equipment correspondingly has led to significant issues and compromised people's safety. In such circumstances, intelligent systems capable of remotely monitoring patients' conditions and providing specialist support would have been enormously beneficial.

Essentially, the trends observed in data production are primarily driven by two key factors: the digital services provided by a multitude of stakeholders from diverse sectors, and their extensive adoption by millions of users globally. This study thoroughly explores this phenomenon by integrating various sources and making two significant contributions: (i) providing informed and up-to-date “guesstimates” of the yearly data production for some of currently top big data entities, and (ii) enabling comparisons among different sectors or data streams, including data production, storage, and transmission.

### 1.2. Prominent big data producers and sources

**Figure 1:**
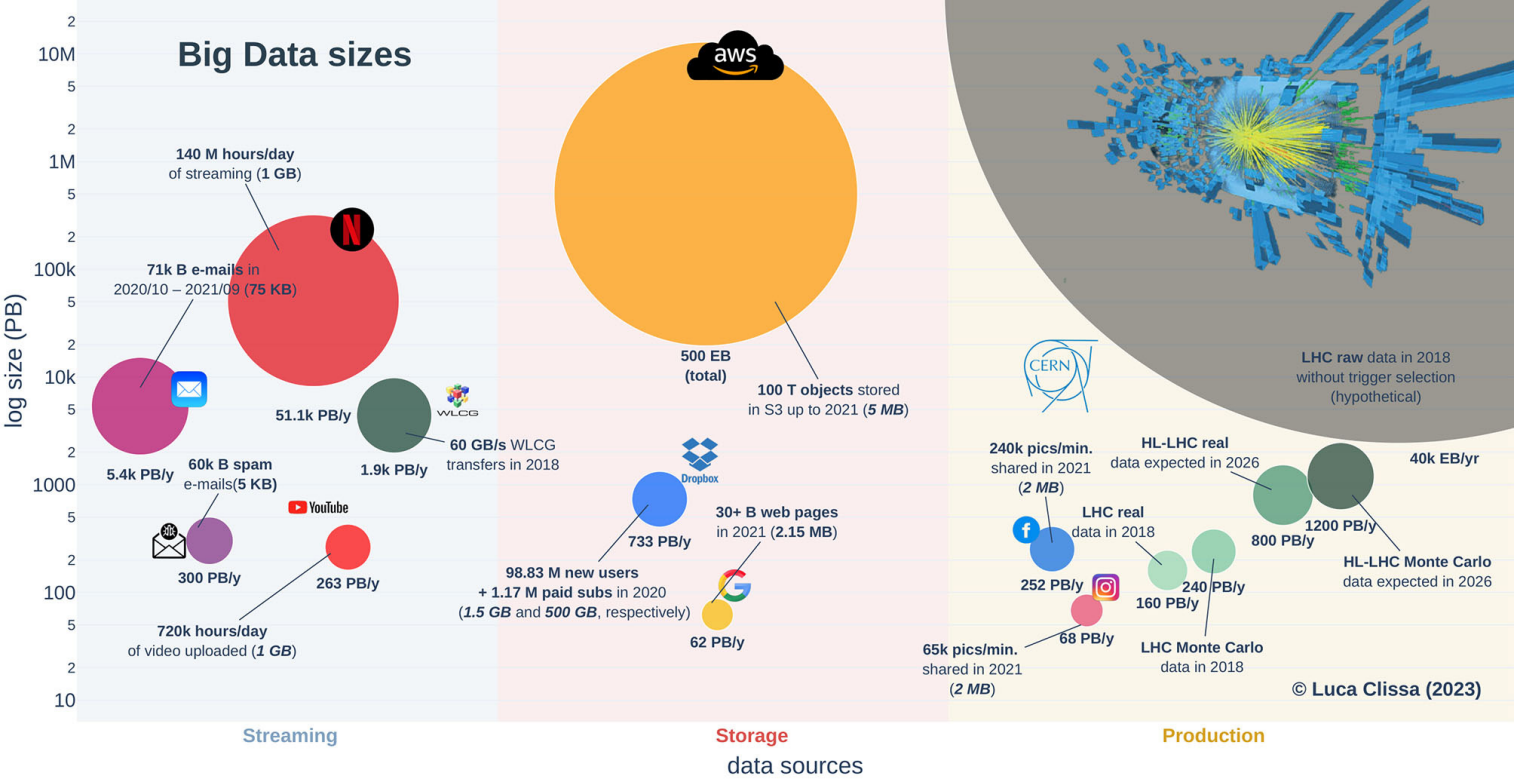
**Big data sizes**. Orders of magnitude involved in different data sources for several big data players. The area of each bubble represents the amount of data streamed, hosted or generated. The accompanying text annotations emphasize the crucial factors considered in the estimation process. Average per-unit sizes are indicated in parentheses, where italic denotes measures derived from reasonable assumptions due to the absence of available references.

The list of organizations contributing to the generation and dissemination of digital data in the modern society is extensive, encompassing tech companies, media agencies, institutions, research centers and more. Conducting an comprehensive survey involving all these stakeholders would be exceedingly challenging, if even impossible. Consequently, this study focuses solely on a subset of these entities and conducts a comparative analysis of their yearly data production. Specifically, various online sources are extensively mined to gather information about the volume of contents produced, hosted or streamed by some of the major players in the field of big data. The corresponding yearly production rates are then derived based on reasonable estimates of unitary sizes for such contents, e.g., the average size of emails or pictures, average data traffic for one hour of video, and so on. Notably, considerations related to storage space are omitted due to the lack of information regarding data management policies, such as data replication and redundancy. Figure 1 illustrates the results of this comparative analysis, while Table 1 summarizes the estimation procedure and the sources of information considered. The reported values are not meant to be pinpoint accurate; rather, they provide a general understanding of the orders of magnitude involved.

**Table 1:** Summary of the estimation process.

	**YouTube**	**Dropbox**	**Facebook**	**Instagram**	**Google**	**LHC data (real1)**	**LHC data (Monte Carlo)**	**HL-LHC data (real)**	**HL-LHC data (Monte Carlo)**
Production unit	720k hours/day video uploads (Dean, 2021b)	100 M new users (1.17 M paid subs) (Dean, 2021a)	240k photos/min uploaded (Domo, 2021)	65k photos/min uploaded (Domo, 2021)	30+ B webpages (De Kunder, 2021; Djuraskovic, 2021)	161 days of data taking (Todd et al., 2018)	1.5 LHC real data	5+ times LHC real data (Aberle et al., 2020)	1.5 HL-LHC real data
Per-unit size	1 GB (Vera et al., 2019)	1 GB (free accounts)
and 400 GB (paid)	2 MB (Adobe, 2021)	2 MB (Adobe, 2021)	2.15 MB (Teague et al., 2021)	1 PB (CERN, 2017)			
Period	2021	2020	2021	2021	2021	2018	2018	2026	2026
	**Amazon S3**	**LHC raw data**	**E-mails**	**Spam**	**WLCG data trasfer**	**Netflix**
Production unit	100 T objects (Barr, 2021)	2400 M particle collisions per second (Grandi, 2017)	71k B mails sent (Statista, Research Department, 2021)	60k B junk mails sent (Statista, Research Department, 2021)	Throughput (s)	140 M hours/day (Domo, 2021)
Per-unit size	5 MB (Hampton, 2021)	1 MB (Grandi, 2017)	75 KB (Tschabitscher, 2021)	5 KB (Baker, 2014)	60 GB (WLCG, 2019)	1 GB (Perry, 2021)
Period	Up to 2021	2018	Oct 20 to Sep 21	Oct 20 to Sep 21	2018	2021

The table reports a recap of the production units and the per-unit sizes considered in the estimation process for different big data sources, along with the corresponding time period.

Despite not being the widely known among the mainstream audience, the CERN community (CERN, 2023b) holds a prominent position in terms of big data *production*. Indeed, the readout electronics of the physics experiments conducted by CERN scientists utilizing the Large Hadron Collider (LHC) (CERN, 2023a) generated roughly 40 ZettaBytes (ZB) of raw data during its last run (2018) (Grandi, 2017). In comparison, Amazon Simple Storage Service (S3) stored over 100 trillion objects until 2021 according to Amazon Web Service (AWS) chief evangelist, Barr (2021). Assuming an average size of 5 MB per object in a representative S3 bucket (for instance, see Hampton, 2021), the total amount of data produced by LHC collisions in one year would exceed the total size of files ever stored on Amazon cloud storage services by approximately one order of magnitude, i.e., 40 ZB against roughly 500 Exabytes (EB), respectively. However, storing the raw readout electronics is currently unattainable with existing technology and budget constraints. Moreover, only a fraction of that data is genuinely relevant for the study of new physics phenomena, making it unnecessary to retain all the information. Consequently, the vast majority of raw data is promptly discarded using hardware and software trigger selection systems, significantly reducing the *recorded* data volume. As a result of this cut, the actual acquisition rate stands at nearly 1 PetaByte (PB) per day (CERN, 2017), equivalent to roughly 160 PB^1^ a year in 2018. In addition to the actual data collected by LHC, physics analyses necessitate the comparison of experimental results with Monte Carlo data, simulated based on current theories, resulting in ~1–2 times^2^ additional data (Grandi, 2017). Furthermore, the CERN community is actively working on enhancing the capabilities of the Large Hadron Collider for the High Luminosity (HL-LHC) upgrade (Aberle et al., 2020). As a consequence, the generated data are expected to increase of a factor ≥5 (Aberle et al., 2020), resulting in an estimated 800 PB of new data each year by 2026. In terms of other renowned big data stakeholders such as Google and Meta, the services they provide generate a yearly data production comparable to the effective figures of LHC, amounting to a few hundreds petabytes.

For instance, the Google search index tracked at least 30 billion webpages in 2021 (Van den Bosch et al., 2016; Indig, 2020; De Kunder, 2021; Djuraskovic, 2021), which gives a total of 62 PB when considering an average page size of 2.15 MB (Teague et al., 2021). Regarding YouTube video uploads, instead, 720 thousands hours of footage were uploaded daily (Dean, 2021b), resulting in roughly 263 PB when assuming an average size of 1 GB (Vera et al., 2019). Similarly, the photos shared on Instagram and Facebook amount to an estimated 68 PB and 252 PB, respectively, given that 65,000 and 24,0000 pictures where shared every minute on these social media (Domo, 2021) and assuming 2 MB as the average picture size (Adobe, 2021). The yearly data production even increases when considering storage services like Dropbox. In 2020, the company reported 100 million new users, 1.17 millions of which were paid subscriptions (Dean, 2021a). Assuming that free accounts utilized 75% of the 2 GB storage available, and that paid accounts occupied 25% of the total 2 TB, the amount of new storage required by Dropbox users in 2020 is ~768 PB.

Apart from the nominal values of generated information, data *streaming* constitutes a significant slice of the big data market. The continuous flow of small- to medium-sized files results in massive traffic when scaled up to millions of users. For instance, *Statista* reports that nearly 131 trillion electronic communications were exchanged from October 2020 to September 2021, comprising 71 trillion emails and 60 trillion spam messages (Statista, Research Department, 2021). Assuming average sizes of 75 and 5 KB for standard (Tschabitscher, 2021) and junk (Baker, 2014) emails, respectively, this leads to an estimated 5.7 EB traffic during the analyzed period, surpassing the amounts discussed so far. Another example of substantial data streaming is represented by Netflix, which operates on an even larger scale. The company's user base has experienced significant growth in recent years, particularly due to changes in daily routines imposed by the pandemic. According to the 9*-th* edition of the *Data Never Sleeps* report by *Domo*, Netflix users consumed 140 million hours of streaming per day in 2021 (Domo, 2021). This translates to a total of roughly 51.1 EB assuming 1 GB of data for standard definition videos (Perry, 2021). Surprisingly, the scientific community also plays an important role in the data streaming context. Indeed, large collaborations comprising thousands of researchers worldwide orchestrate the LHC experiments at CERN. Consequently, the data collected at CERN are continuously transferred via the Worldwide LHC Computing Grid to fuel innovative research (WLCG, 2023). For example, a throughput of 60 GB/s was achieved in 2018 (WLCG, 2019), resulting in a yearly projection of 1.9 EB, which is close to half of the global email traffic and only one order of magnitude lower than Netflix usage.

## 2. Discussion

The data production rate is currently at its peak, and this trend is expected to continue growing in the coming years. Conducting an exact comparison of the information generated by various organizations contributing to this surge is extremely challenging, if not practically unfeasible. This study aims to offer reasonable indications of the latest orders of magnitude of yearly data production for some of today's main players in the realm big data. However, it is important to note that the lack of official sources prevents precise estimations of the big data volumes produced by individual organizations. For the same reason, the amount of storage space occupied by these organizations is not considered in this study, as it would require more detailed information about their data management policies.

A fundamental observation that emerges from this survey is that streaming data already account for a significant portion of the big data market, and this is expected to persist in the future due to the growing adoption of smart everyday objects capable of generating and sharing data.

Additionally, a noteworthy finding is that the experimental data collected by the scientific community play a substantial role in the big data phenomenon. Specifically, the data volumes generated by nuclear physics experiments conducted at CERN are comparable to the traffic experienced by some of the most prominent commercial players, such as Google, Meta, and Dropbox.

## Author contributions

LC: Conceptualization, Data curation, Investigation, Methodology, Project administration, Validation, Visualization, Writing—original draft, Writing—review and editing. ML: Validation, Writing—review and editing. LR: Writing—review and editing.

## Footnotes

^1^LHC registered 161 days of physics data taking in 2018 (Todd et al., 2018).

^2^A factor of 1.5 was adopted here for the bubble plot.
